# Epigenetic meets metabolism: novel vulnerabilities to fight cancer

**DOI:** 10.1186/s12964-023-01253-7

**Published:** 2023-09-21

**Authors:** Domenica Scumaci, Qingfei Zheng

**Affiliations:** 1 Research Center On Advanced Biochemistry and Molecular Biology, Magna Græcia University of Catanzaro, 88100 Catanzaro, Italy; 2 Department of Experimental and Clinical Medicine, Magna Græcia University of Catanzaro, 88100 Catanzaro, Italy; 3 Department of Radiation Oncology, College of Medicine, The Ohio State University, Columbus, OH 43210 USA; 4 Center for Cancer Metabolism, James Comprehensive Cancer Center, The Ohio State University, Columbus, OH 43210 USA; 5 Department of Biological Chemistry and Pharmacology, College of Medicine, The Ohio State University, Columbus, OH 43210 USA

**Keywords:** Metabolic reprogramming, Histone post-translational modification, Histone non-enzymatic covalent modification, Cancer onset and progression

## Abstract

Histones undergo a plethora of post-translational modifications (PTMs) that regulate nucleosome and chromatin dynamics and thus dictate cell fate. Several evidences suggest that the accumulation of epigenetic alterations is one of the key driving forces triggering aberrant cellular proliferation, invasion, metastasis and chemoresistance pathways. Recently a novel class of histone “non-enzymatic covalent modifications” (NECMs), correlating epigenome landscape and metabolic rewiring, have been described. These modifications are tightly related to cell metabolic fitness and are able to impair chromatin architecture. During metabolic reprogramming, the high metabolic flux induces the accumulation of metabolic intermediate and/or by-products able to react with histone tails altering epigenome homeostasis. The accumulation of histone NECMs is a damaging condition that cancer cells counteracts by overexpressing peculiar “eraser” enzymes capable of removing these modifications preserving histones architecture. In this review we explored the well-established NECMs, emphasizing the role of their corresponding eraser enzymes. Additionally, we provide a parterre of drugs aiming to target those eraser enzymes with the intent to propose novel routes of personalized medicine based on the identification of epi-biomarkers which might be selectively targeted for therapy.

Video Abstract

**Supplementary Information:**

The online version contains supplementary material available at 10.1186/s12964-023-01253-7.

## Introduction

Chromatin is a macromolecular complex of DNA and proteins that allow the packaging of the whole genome into nucleosomes. Nucleosome is the functional unit of chromatin and comprises four pairs of histones wrapped around by DNA. Histones are composed by globular domains located within the nucleosome and flexible “tails” that protrude from the nucleosomal structure. Histone tails undergo a parterre of canonical post-translational modifications (PTMs) that are deposited by writer enzymes, removed by eraser enzymes and translated into specific functions by reader enzymes [1]. Recently a novel class of histone PTM have been described: these modifications are classified as non-enzymatic covalent modifications (NECMs) and comprise a number of covalent modifications whose reactions are not enzymatic and are influenced only by the reagent concentration. A body of evidence suggests that NECMs are tightly related to cell metabolism [2, 3]. Metabolic pathways enclose the whole enzymatic reactions that cells use to produce ATP via glucose, lipid and amino acid catabolism and build cellular components through the anabolic reactions. Metabolic fitness accounts for the production of metabolites that can freely diffuse through the nuclear pore becoming in metabolic homeostasis with the cytoplasm [4]. In the nucleus, these metabolites might direct the activity of histones modifiers (e.g., IDH2 and acetyl-CoA) or in the case of NECM might directly react with histones tails altering chromatin landscape [5, 6]. In cancer cells, the correlation between epigenome homeostasis and metabolic rewiring is a demanding field of investigation that urge to be deepened [2, 3]. Metabolic rewiring is a hallmark of cancer. Transformed cells rewire their metabolism to meet the energetic requirements sustaining proliferation, survival, and invasion [7–12]. The high energetic flux increases the concentrations of metabolites that are potentially harmful to cells and might compromise genomic information, impairing their proliferative potential.

In this context, cancer cells have evolved and strengthened several strategies, including metabolite efflux, metabolite scavenging, and adduct removal to prevent the accumulation of NECMs preserving the integrity of the chromatin architecture. Unveiling the mechanisms underlying the dynamic nature of NECMs may result crucial to comprehend the fitness of metabolically dysregulated cells and identifying novel therapeutic targets aiming at improving cancer therapy. This manuscript aims to explore the main NECMs, pointing out the strategy that cancer cells adopt to reduce their negative effects on chromatin architecture. Moreover, in a perspective context, we provide an overview of the current approach aiming to counteract these mechanisms of escape for the selective targeting of cancer cells.

## Main categories of NECMs

### Overview on NECMs

Carboxylate groups, that are able to interfere with histone tails altering chromatin topology, are those derived from acetyl-CoA [11, 12], malonyl-CoA [13, 14], benzoyl-CoA [15], lactyl-CoA [16], lactoylglutatione [17] and thiolactone [16]. In the nuclear compartment, the carboxylate group are inert, due to its low electrophilicity, but it can become a good electrophile when enzymatically converted into derived thioesters or anhydrides and thus capable of acylation. Acylation might be considered enzymatic when catalyzed by a specific enzyme such us histone acetyltransferase. When the acyl group derives from malonyl-CoA, benzoyl-CoA, lactyl-CoA, lactoylglutatione and thiolactone the modification is generally named acylation. Most recently, several authors provide evidence that acylation could be also the result of a “non-enzymatic reaction” particularly when the levels of reactive acyl-ester are dramatically increased in response to the activation of peculiar metabolic pathways [13].

The majority of novel histone PTMs are classified as short-chain Lys acylations. These modifications are functionally similar to Lys acetylation (Kac), a well-characterized histone modification that consists in the deposit of the acetyl group on the on the ε-amine group of Lys residue. Acylations occur also to the ε-amine group of Lys, but exhibit a distinct structural proprieties characterized by peculiar hydrocarbon chain length, hydrophobicity and charge [14].

Although acylation might be non-enzymatic, the removal of the acyl group is always a fine-tuned enzymatic reaction catalyzed by a member of the histones deacetylases class (HDACs) or sirtuin family enzymes [18, 19].

**Fig. 1:**
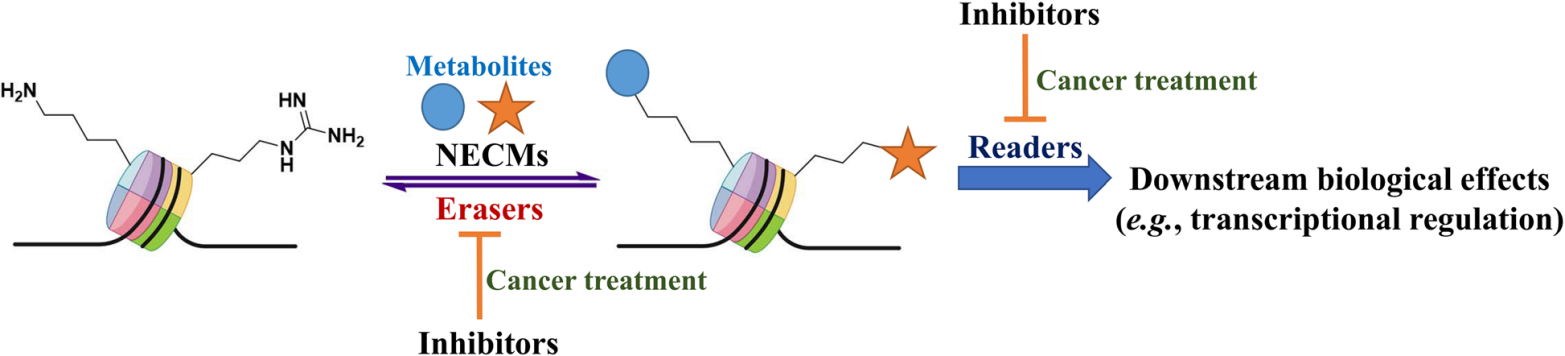
The figure is representative of the mechanism that directs histones’ non-enzymatic modification. In cancer cells, high metabolic flux accounts for the overproduction of metabolites and waste products that under stress conditions can be activated in thioesters and react with histones tails in a non-enzymatic manner. These modifications might impair nucleosome architecture. Cancer cells counteract these conditions by overexpressing eraser enzymes. These erasers have a crucial role in cancer progression and might be precisely fought in a translational setting

Acylation occurs at the ε-amino group of lysine residue and resulting in the loss of a positive charge, leads to the de-condensation of chromatin (euchromatin). The dynamic change of heterochromatin to euchromatin accounts for a transcriptionally activated state. Indeed several evidences suggest that acylation pattern exerts the activation of the transcription similarly to the acetylation counterparts. Overall, acylation is related to numerous physiological functions, such as spermatogenesis, tissue damage, metabolic injury and metabolic homeostasis [14–23]. Moreover, involving a multitarget functional group, acylation is mutually exclusive with other histone PTM exhibiting opposite effects. For example, the removal of acylation provides an open site for methylation that correlate with a repressive transcription state. The removal of acylation groups is specifically ascribed to peculiar histone deacetylase (HDAC): sirtuin-family HDACs exert their activity on succinyl, malonyl and glutaryl groups [24–26]. SIRT6 is active on long-chain fatty acyl modifications [27] and various sirtuins specifically account for the removal of short-chain fatty acyl modifications (propionyl, butyryl, crotonyl) [26, 27]. Likewise acetylation, acylation might be recognized by acetylation readers modules, such as BRD4 and BRD9, that decode peculiar downstream signaling pathways [28–30]. The class of Acylation modification has been recently enlarged and includes formylation [31, 32], propionylation [33–36], butirylation [33–36], crotonylation [35, 37], 2-hydroxyisobutyrylation [38], succinylation [39, 40], malonylation [40], and glutarylation [23, 26]. The general mechanism of histone NECMs depositing, removing and reading is reported as Fig. 1.

### Crotonylation

Histone crotonylation raises a huge interest owing to the evidence that this histone mark was found on several lysine residues of the histone linker H1, in regions specifically associated with a transcriptionally active state. Histone crotonylation is regulated by the intracellular levels of crotonyl-CoA, whose amount depends on genetic and environmental events, such as the availability of extracellular sodium crotonate, [26, 27]. Recently, numerous authors focused their attention on the mechanism that leads to fluctuation in crotonyl-CoA levels. Crotonyl-CoA is a crucial hub of metabolic networks connecting cytosolic and mitochondrial pathways, whereby its level results from the cross-talk between lipid and amino acid metabolism [41, 42]. The levels of Crotonyl-CoA increase during beta-oxidation and metabolism of lysine and tryptophan. Therefore, it is plausible that metabolic homeostasis governs the fluctuation of crotonyl-CoA that ultimately fuels histone crotonylation [13]. In normal conditions, the concentration of this compound is 3-fold lower than the amount of acetyl-CoA, which means that histone acetylation is much more abundant than crotonylation.

Since Acetyl CoA and crotonyl CoA competes for the same Lysine residues, the main modulator of histone crotonylation is the availability of acetyl-CoA. Indeed, it has been reported that the reduction of acetyl-CoA by ATP Citrate Lyase (ACLY) or Pyruvate Dehydrogenase Complex (PDC) depletion, decreases H3K18 acetylation fueling H3K18 crotonylation [43, 44]. Conversely, the diminution of Acyl-CoA Synthetase Short Chain Family Member 2 (ACSS2) activity, which concomitantly catalyzes the biosynthesis of crotonyl-CoA and acetyl-CoA, induces downregulation of crotonylation at gene promoters affecting genomics expression. The major substrate for crotonyl-CoA synthesis is crotonate. Sabari et al. reported that the medium implementation with short-chain fatty acid (SCFA) including crotonate, to grown HeLa S3 cells, induces high levels of crotonyl-CoA and a consequent increase of H3K18cr [43]. Intriguingly, Tan and colleagues suggested that gut microbiota account for the production of crotonate, through a fermentative pathway overall increasing the amount of SCFAs. SCFAs bypass the intestinal membrane reaching the tissues where they are activated to acyl-thioesters that lastly fuel acylation reaction [45].

### Formylation

Histone Formylation has been reported as a non-enzymatic histone PTM occurring under drastic conditions such as oxidative and nitrosative stress. The impact of histone formylation in gene expression is not clearly elucidated although in eukaryotes, protein formylation is ubiquitous [46]. Histones formylation of chromatin in a novel point of crosstalk between epigenetics and metabolism. The existence of deformylases enzyme [47] as well as receptors for formyl-binding proteins [48] account for the subsistence of peculiar metabolic and signaling pathways able to respond to damaging stimuli.

Formylation, mediated by Acyl phosphates, is described as a further route of non-enzymatic reaction either in prokaryotes or in eukaryotes [31]. The most likely hypothesis is that formyl donors could derive from 3’-formyl phosphate, a highly reactive metabolite generated during the oxidation of the 5’-deoxyribose in the damaged DNA. A further source to generate formyl-lysine might involve formaldehyde through the oxidation of the carbinol-amine intermediate during the reaction with the amine side chain of lysine. Formaldehyde is a by-product of several cellular processes including nucleic acid demethylation and biosynthesis of purines, thymidine and specific amino acids. Interestingly some authors proposed also that formaldehyde might be generated as a waste of the histone demethylation process [49–51].

Formylation, likewise to the previously discussed acylation reaction, is a crucial epigenetic regulator in mammalian cells based on its chemical similarity to histone lysine acetylation. Formylation, occurring on lysine residues, competes with acetylation and methylation. This type of interference, and at times cross-talk, contributes to the pathophysiology of oxidative and nitrosative stress [31].

### Propionylation

Propionylation of histone lysine was detected in mammalian cells and is regarded as a mark of active chromatin. In the landscape of histone acylation, propionylation is structurally similar with acetylation and probably, at the light of this analogies, these two epigenetic marks could overlap their functions as explained by Simithy et colleagues [13].

Propionyl-CoA is the activated thioester of propionic acid. Propionic acid (PA) is generated by anaerobic bacteria through carbohydrates fermentation in the intestinal lumen. It is the most abundant component of short-chain fatty acids (SCFAs). SCFAs are crucial metabolites of the intestinal cells since they absolve several functions including counteraction of pro-inflammatory intermediate generation, maintenance of the acidic gut environment, preservation of the integrity of the epithelial membrane and regulation of the proliferation of pathogens agents [52]. In this context, SCFAs exhibit a crucial role as a source of putative epigenetic markers [52].

Propionyl-CoA might be also generated via metabolic pathways being an intermediate of odd-carbon fatty acids, cholesterol and essential amino acids catabolism. The levels of propionyl-CoA is modulated by the activity of propionyl-CoA carboxylase (PCC), a biotin-dependent enzyme, that metabolizes propionyl-CoA to methylmalonyl-CoA. In this context, the over-activation of specific metabolic pathways might induce a fluctuation on its levels lastly affecting the levels of histone ropionylation. A milestone work of Kebede and colleagues proposed histone propionylation as a mark of active chromatin unveiling that the large part of active genes in mouse livers exhibit multiple acylation marks and providing evidence that H3K14pr correlated with a higher transcriptional output. Notably, the authors propose that histone propionylation is an intriguing candidate linking metabolic fitness with chromatin landscape and focused on propionyl-CoA carboxylase, the enzyme able to degrade propionyl-CoA, as a crucial target to modulate propionyl-CoA levels and lastly propionylation [21]. Accordingly, with these findings, Liu and colleagues observed iper-propionylation in the leukemia cell line U937 pointing up that H3K23 was a distinctive mark of highly proliferating cells. In addition, they proposed, for the first time, that the propionylation was governed by the aberrant accumulation of propionyl-CoA arising from either over-activation of propionyl-CoA synthetase or down-regulation of propionyl-CoA carboxylase [34].

### β-hydroxybutyrylation

An additional form of histone acylation is Lysine β-hydroxybutyrylation (Kβhb). This type of modification is driven by ketone bodies generation under restricted nutrient conditions.

In the liver, during prolonged intense exercise or under nutrient deprivation, fatty acids are catabolized in an alternative pathway named ketogenesis. The generation of ketone bodies is dramatically increased in pathological conditions such as diabetes. Ketogenesis leads to the production of butyrate that is activated to the correspondent CoA. The activation of butyrate to β-Hydroxybutyryl-CoA (βhb-CoA) involves the enzyme acyl-CoA synthase short-chain family member 2 (ACSS2) that converts the short-chain fatty acids to the activated thioester [53, 54]. During fasting, histone Kbhb dramatically increases on H3K9 modulating a cluster of genes which induce starvation response [55]. Conversely, the inhibition of ACSS2, decreasing the availability of βhb-CoA, greatly affects fasting response [56].

β-Hydroxybutyryl-CoA (βhb-CoA) is also a transient intermediate in the last reaction of fatty acids β-oxidation, moreover, it could derive from the bacterial fermentation of butyric acid, and the catabolism of lysine and tryptophan [55]. Within this framework, Kbhb, induced by fatty acid β-oxidation, seems to be determinant for the regulation of genes implicated in lipid metabolism [57].

Notably, the removal of β-hydroxybutyryl marks, by de-β-hydroxybutyrate, is also a crucial event. Zhang and colleagues have established that SIRT3 exhibits the ability to erase Kβhb from H3K9bhb underling its peculiar de-β-hydroxybutyrylase activity [58]. Alongside Huang and colleagues provided evidence that also HDAC1 may acts as Kbhb erasers [59]. The ability of these enzymes, in governing histone dynamic acylations, unveil their crucial role as a master regulator of the mechanism connecting metabolism to gene expression.

### Succinylation

Several studies demonstrated that lysine succinylation is a novel histone mark in eukaryotes [60]. The Succinyl-group reacts with the ε-amine of lysine with a mechanism that might be enzymatic or non-enzymatic.

The reaction leads to a change in the charge state of residues from + 1 to − 1 at physiological pH. This charge change induces a net charge of + 2, with an increase of residues hydrophobicity, consequent destabilization of the nucleosome and hinder of histone interactions [14, 61]. Overall, succinylation mimics acetylation by directly altering the chemical architecture of nucleosome and thus chromatin packaging.

Several evidences suggest that succinylation is mainly a non-enzymatic reaction, and then directly related to the abundance of succinyl-CoA [13]. In this framework, authors concluded that succinylation as well as malonylation and glutarylation, are most susceptible to non-enzymatic acylation by rule of its intrinsic reactivity. In addition, they suggest that acyl transferase preferentially works with acetyl-CoA rather than with other acyl coenzymes suggesting that the size of acyl groups might be also a discriminant factor.

Ultimately it is suggested that succinylation preferentially occurs at the C-terminus hypothesizing that histones peculiarly undergo enzymatic reaction at N-terminus and non-enzymatic reaction at C-terminus exhibiting in their landscape a different reactivity [62, 63].

In this context, it is also reported that lysine histone acetylase (HAT) exhibits the ability to bind, with higher affinity, linear, smaller and charge-neutral derivatives such as acetyl-coA [14]. Conversely, the acidic acyl group (malonyl-CoA, succinyl-CoA, and glutaryl-coA) and larger derivatives (β-hydroxybutyryl-CoA, benzoyl-CoA) are less affine with the enzymatic catalytic sites and thus more prone to non-enzymatic reaction [14].

### Malonylation

Malonyl-CoA is an acidic acyl group, negatively charged with electrophilic proprieties [3]. These characteristics confer a low susceptibility to enzymatic reaction enforcing the notion that malonylation is exclusively a non-enzymatic reaction. Histone malonylation has been identified through an MS-based approach in yeast on core histones at H2AK119, H2BK116, and H3K56, [35]. Interestingly, H2AK119 modification is important as it is the first example of cross-talk between non-enzymatic acylation and enzymatic phosphorylation PTM. Indeed Ishiguro and colleagues reported that H2AK119mal impairs the Bub1 kinase hampering the interaction with the proximal H2AS121 (H2AT120 in humans), and preventing its phosphorylation [14]. The lack of phosphorylation on H2AS121 blocks the binding of Shugoshin proteins causing defects in chromosome segregation [64]. Evidence suggests that lipid metabolism generates the large part of Malonyl-CoA that might fuel histone acylations. Malonyl-CoA might be generated during the first step of de novo fatty acid biosynthesis. The reaction is catalyzed by acetyl-CoA carboxylase (ACC) which adds a molecule of carbonic acid to a molecule of acetyl-CoA forming malonyl CoA. Pools of malonyl-CoA are also supposed to be generated within the mitochondrial matrix, by the action of propionyl-CoA carboxylase and within peroxisomes during the β-oxidation of odd chain-length dicarboxylic acids [65]. Levels of histone malonylation are directly related to the amount of Malonyl CoA, suggesting that lipid metabolism has a key role in modulating this modification.

### Glutarylation

Lysine glutarylation (Kglu) was found as histone marks of several lysine residues on human core histones. Histone Kglu impacts chromatin architecture and therefore induces alteration of transcription and aberration in cell-cycle regulation, DNA damage pathway, and telomere silencing. [13] The precursor of glutarylation is Glutaryl-CoA. A thioester derivate of glutaric acid and coenzyme A. The main source of Glutaric acid is lysine and tryptophan catabolism. These catabolic routes occur within mitochondria, where it is also allocated the glutaryl-CoA metabolism [66]. The master regulator of the amount of Glutaryl-CoA is the Glutaryl-CoA dehydrogenase (GCDH) that converts glutaryl-CoA to crotonyl-CoA through oxidative decarboxylation. Interestingly the GCDH KO induce in mice increased levels of Kglu [45]. The accumulation of Glutaryl-CoA into mitochondria induces TCA cycle dysfunction [67] affecting mitochondrial energy metabolism and leading to ageing as well as ageing-related diseases, such as cancer and neurodegeneration [68]. Similarly, to other acyl groups, glutaryl-CoA can directly induce non-enzymatic Kglu [23]. The level of Kglu is mainly affected by the amount of glutaryl-CoA that might increase when cells are forced to use amino acids as carbon sources [69]. The enhancement in glutaryl-CoA concentration might increase the level of Kglu in vivo [14]. Histone glutarylation has been reported as a novel histone acylation [70]. Although some authors suggested KAT2A as the enzyme able to mark H4K91Glu [71], a writer for this modification has not been identified yet. Histone glutarylation changes the positive charge of lysine in a negative site inducing overall chromatin decompaction and impairing the modification on proximal positive sites [72].

### Lactylation

Lactylation is a recently discovered protein acylation occurring on lysine residues [15, 17, 72, 73], where the acyl donors can be either L- or D- lactate [74]. Lactylation was first identified as an epigenetic marker on histones, which has different temporal dynamics from histone acetylation and regulates gene expression [17, 72, 75] as a new link between cellular metabolism (e.g., glycolysis) and epigenetics. In fact, lactate has long been considered a “dead-end” waste product of anaerobic glycolysis [76] before the discovery of lactylation. As a protein PTM donor, lactate actually possesses more significant pathophysiological roles than being a tissue pH regulator [77]. Even though acyltransferases [such as the histone acetyltransferase (HAT), p300, and YiaC] have been shown to act as the possible writer enzymes for lactylation [16, 72, 78], lactyl-CoA is able to directly modify lysine residues to lactyl-lysine in a non-enzymatic manner. Moreover, lactoylglutathione has been proven to serve as another major lactyl donor for non-enzymatic protein lactylation [73].

### Glycation

Glycation is one of the most well-studied non-enzymatic PTMs occurring on protein residues, mainly including lysines and arginines [79]. Glycation is attributed to the Maillard chemistry occurring between reducing sugar molecules and nucleophiles of proteins (such as lysine, arginine, cysteine, etc.). For instance, the aldehyde group of glucose is able to spontaneously react with the primary amines of lysines in diverse proteins (including histones), resulting in the generation of Schiff bases and Amadori products [80]. Due to the further oxidation and rearrangement, these early-stage products are able to be converted to advanced glycation end products (AGEs) that contain complicated aromatic ring structures (such as glucosepane) [81]. In addition, other representative reducing sugars (including ribose, fructose, methylglyoxal and glyoxal) can also induce glycation on cellular proteins [82–85]. We and other labs reported that methylglyoxal-induced histone glycation is associated with human disease states (especially cancer) and can regulate the chromatin architecture through charge effects and covalent crosslinking, thereby influencing gene transcription [84–88]. Moreover, other research work has also shown that the glycation on non-histone proteins (such as NRF2 and KEAP1) is able to either positively or negatively influence cancer progression through different mechanisms [89–91]. Given the nature of reducing sugars as essential cellular metabolites, glycation may serve as a new link between metabolism and cell signal transduction.

### Monoaminylation

Monoaminylation is a ubiquitous PTM that has been identified on diverse proteins. Recently, the monoaminylation of histone H3 has been characterized as a new epigenetic marker, which play a role in regulating gene transcription [92]. The specific site of monoaminylation on histone H3 is its fifth residue in the N-terminus, i.e., glutamine (Q). Two distinct monoaminylations on H3Q5 were first identified, where serotonin and dopamine serve as donors, respectively [93, 94]. It has been shown that transglutaminase 2 (TGM2) is the writer enzyme that can install these two monoaminylations specifically to H3Q5 through transglutamination [93–95]. Till now, the readers that can recognize H3Q5ser or H3Q5dop have not been discovered, while a lot of evidence showed that histone monoaminylation could significantly affect gene transcription indirectly by influencing the other readers targeting nearby histone PTMs (e.g., H3K4me3) [93, 94].

Unlike other NECMs that can usually non-specifically modify multiple sites of one protein, histone monoaminylation solely occurs on H3Q5, because it is enzymatically installed by TGM2. However, more and more evidence shows that TGM2-mediated monoaminylation has many similarities as NECMs [95, 96]. Recently, we applied chemical biology approaches to understand the dynamic control of histone monoaminylation and unexpectedly discovered that the installation, removal, and replacement of this modification are all mediated by the single enzyme, TGM2. The biochemical mechanism of this novel regulation is attributed to the formation of a reactive thioester complex between TGM2 and H3 that can be attacked by nucleophiles (such as serotonin and dopamine). Based on this unique enzymology, we predicted and identified an unreported histone monoaminylation, H3Q5 histaminylation (H3Q5his), and found that this new epigenetic marker promotes neural rhythmicity through epigenetic regulations [92]. This NECM-like characteristic of TGM2-mediated monoaminylation makes it to be a cellular microenvironment-driven epigenetic modification, which means that the reaction fate of TGM2-activated H3 is mainly determined by the donor types in the microenvironment.

## Targeting histone non-enzymatic covalent modifications

Recently the targeting of histone non-enzymatic covalent modifications has become an intriguing field of investigation. Although the deposit of NECMs might be a non-enzymatic reaction, the removal of these histone marks is always an enzymatic reaction catalyzed by a peculiar enzyme that selectively removes the modification. In this scenario, the modulation of the “eraser enzyme” might represent a strategy to precisely target cancer cells exhibiting the aberrant activation of specific metabolic routes. Here we propose an excursus on the enzyme implicated in the removal of NECMs pointing out drugs and strategy that exert anti-cancer activity.

### Crotonylation targeting

The removal of the crotonyl group is mediated, in Mammalia, by the NAD^+^-dependent class III Histone deacetylates, sirtuin 3 (Sirt3). The inhibition of HDAC3 by histone deacetylase inhibitors, such as Vorinostat, Trichostatin, SHAH or MS275 impairs HDCR activity affecting the levels of crotonylation [22].

Histone kcr is implicated in several cellular functions spanning from health to disease settings. The mechanism of Kcr, as well as the modulation of its levels in response to biological processes, are still debated. Accumulating evidence connects this histone mark with proliferation, DNA damage and ageing-associated mechanisms [97–100]. Histone Kcr were found dysregulated in several cancers, including, stomach, liver, kidney, thyroid, esophagus, colon, pancreas and lung carcinomas [97, 98]. In hepatocellular carcinoma (HCC), it was found that high levels of Kcr, induced by the siRNA interference of histone deacetylases (HDACs) or HDAC inhibitors, decrease cancer cell motility and proliferation [101]. Sirt3 is the histone deacetylase that exhibits a distinctive activity in the Kcr marks removal, and it is reasonable to believe that inhibition of SIRT3 might exert remarkable anti-tumoral effects (also) by modulating histone kcr levels.

SIRT3 has a crucial role in carcinogenesis, resistance to chemotherapy, cancer metastasis, and the regulation of metabolic reprogramming of cancer cells [102, 103]. The expression of SIRT3 has been proposed as a putative early marker of cancer onset and as an independent criterion to stratify patients prone to develop chemoresistance or metastasis [60].

Remarkable findings report that SIRT3 regulate cancer metabolism rewiring by modulating the acetylation of several enzymes sustaining glycolysis and through the activation of the AMPK/PPAR pathway that, triggering FA synthesis, ultimately promotes cancer metastasis. At the light of this evidence, it seems reasonable that the inhibition of SIRT3, in the context of histone non-enzymatic acylation, might exert a dual role, either by controlling the levels of histone crotonylation, as well as by modulating the activity of a variety of enzymes governing metabolites availability.

In the last decade, several strategies for the targeting of SIRT3 have been implemented. The most straightforward approach consists in the docking of molecular analogues able to compete with the physiological substrate.

The most active Sirt3 selective inhibitors are two independently developed small-molecule: 4’‐bromo‐resveratrol( (4‐BR), and the 8-mercapto-3,7-dihydro-1H-purine-2,6-dione scaffold. The 4’-BR exhibits along with a remarkable inhibition of SIRT3 the ability to interfere with metabolic rewiring in melanoma cells impairing their proliferation potential [104]. The 8-mercapto-3,7-dihydro-1H-purine-2,6-dione scaffold, whose structure was derived using a molecular docking approach, exerts a dramatic inhibition on the isolated enzyme but its activity needs to be further validated by functional data [105].

Several other molecules such us BZD9Q [106], Cambinol [107], NƐ-acyl-lysine analogues [108], 2-methoxyestradiol (2-ME) [109] and Butyrate [110] have been proposed as SIRT3 inhibitors although their mechanisms of action remain still unclear. In addition, some molecules affect SIRT3 levels by interfering its own expression. For example, Albendazole, an anti-helminthic drug, exhibits an off-target effect by promoting SIRT3 degradation and thus cancer cell death [111]. The 3-O-chloroacetyl-gagamine (A671) impairs SIRT3 transcription and elicits anti-proliferative effects in T-lymphoma and erythroleukemia cells [112].

Being SIRT3 a histone deacetylase NAD^+^ dependent, a further strategy to counteract the deacetylase activity consists in hampering the binding of its cofactor [112]. In this context, EX-527 [113], which induces a rearrangement of the NAD^+^ pocket and LC-0296 [114] which acts as NAD^+^ competitor, seem to hold promising anti-cancer effects.

### Formylation targeting

Regarding the “de-formylation” process, Mecclure and colleagues have speculated on the ability of HDAC6 to remove the acyl group pointing out its efficiency in removing the “formyl groups” [115].

The de-formylase specificity of HDAC6 might have a physiologic relevance either in normal conditions then, much more intriguingly, in damaging conditions [116].

Histone deacetylases (HDACs) are a Zn2^+^-dependent enzymes that have a pivotal role in several cellular processes including microtubule dynamics and apoptosis. High levels of HDAC6 have been associated with several hematologic malignancies. Therefore, the development of HDAC6 inhibitors as anti-cancer agents has become a strategic field of investigation. The general approach for HDAC targeting implies the design of molecules able to impound the zinc-binding group [117]. Following the development of the first compound, tubacin [118] whose synthesis resulted wearing and poor, the drug tubastatin was found highly active, selective and able to elicit apoptosis and revert malignant phenotype [119, 120]. More recently, Gajendran and colleagues proposed the molecule JBI-097 as a strong and distinctive inhibitor of the HDAC6 enzymatic pocket. The authors, using a cell-based setting, demonstrated that the molecule exhibits a dramatic anti-proliferative profile compared to precursor compounds [121].

### Propionylation targeting

The most likely enzyme involved in the removal of the propionyl group is SIRT2, a sirtuin NAD^+^ dependent with a crucial role in preserving chromatin architecture [122, 123]. SIRT2 explicates its activity on canonical substrates acting preferentially on long-chain acyl groups. The enzyme also localizes in the cytosol, where it regulates cell division and proliferation [122–124] by deacetylating microtubular proteins such as α-tubulin [125, 126]. Generally, SIRT2 is reported as a tumour suppressor [127] however it has been also reported that its downregulation results in anticancer effect in a model of human breast cancer [128]. Although, in the last decade several SIRT2 inhibitors [128–130] have been proposed, the largest part exhibits a poor specificity for isoenzyme. Recently, Nielsen and colleagues reported a method based on “substrate-mimicking” to efficiently design selective inhibitors. The method led to the identification of thioamide- and thiourea-containing sirtuin inhibitors acting at nanomolar concentration. Although the molecules need to be further characterized, the method might represent an valuable tool for further development of molecules aiming at selectively targeting sirtuin deacetylase isoenzymes [130].

### β-hydroxybutyrylation targeting

The modulation of the β-hydroxybutyrylation (Kbhb) dynamic, through the inhibition of the enzyme, deputed the removal of Kbhb, is a crucial event correlating metabolic homeostasis and epigenetic landscape. The “erasers” of histone Kbhb are predominantly the zinc-dependent HDAC1 and HDAC2 and the NAD-dependent SIRT3 [131].

Although it seems that the activity of SIRT3 overlaps between Kbhb and kkrt, it is reported that the enzyme can discriminate acylation based on the site of modification; indeed SIRT3 exhibits mark-selective activity for histone de-β-hydroxybutyrylation, preferring H3 K4, K9, K18, K23, K27, and H4K16, but not H4 K5, K8, K12 [58].

The most striking observation is that Kbhb leads to the generation of two enantiomers that are discriminated by SIRT3. It was established that R-β-hydroxybutyrate is a metabolite of ketone bodies, which increases in the blood during fasting, starvation, or prolonged intense exercise; conversely, S-β-hydroxybutyrate increases in fed conditions. The deposit of the peculiar histone marks is dependent on the abundance of the enantiomer and modulates the expression of enzymes involved in fasting or fed response. Here the authors demonstrated that SIRT3 preferentially deacetylates the S-enantiomer enforcing the notion that metabolic homeostasis influences the epigenetic landscape and regulates the expression of genes governing metabolic fitness [131].

Although several deacetylases unveil in vitro the ability to erase β-hydroxybutyrylation, only HDAC1 and HDAC2 exhibit this activity in a cells-based context. In the last few decades, since HDACs emerged as a crucial therapeutic target either in hematological malignancies or in solid cancer, several drugs have been investigated and developed for HDAC targeting [132]. While the variety and the number of structures are considerable, a large part of HDAC inhibitors exerts a low ability to discriminate isoenzymes [133]. A recent study reported that Chidamide (CS055) elicits a selective activity toward HDAC1 at micromolar concentration [134].

Similarly, in the HDAC2 setting, the most convincing molecules, inhibiting the enzyme at nanomolar concentration, are Santacruzamate A, active in hepatocellular carcinoma [135] and the monoterpenes Thujaplicins, active in colon prostate and pancreas cancer [136].

### Succinylation targeting

The eraser of histone succinylation is SIRT5, [25]. Importantly, its loss of function impairs the activity of complex II (succinate dehydrogenase [SDH]) and fatty acid β-oxidation, suggesting that succinylation is not only an epigenetic mark but also an allosteric modulator of mitochondrial enzymes [25].

Alongside SIRT5, also SIRT7 seems to have an important role in histone succinylation [25, 137]. SIRT7 has been recently identified as a histone desuccinylase that functionally relates chromatin architecture with the poly-ADP-ribose polymerase (PARP) 1-dependent DNA damage response. Smestad and colleagues have suggested that chromatin hyper-succinylation, following Sirt7 depletion, interferes with DNA repair activities and sensitizes cancer cells to genotoxic agents impairing their survival [138]. These findings imply that lysine succinylation modulates cell metabolism working on several layers of regulation and suggest that Chromatin succinylation may be crucial both for the regulation of genome-wide transcription and DNA repair activities.

Growing evidence suggests that succinylation directs oncogenic signaling by altering redox homeostasis in response to metabolic state. High levels of histone succinylation were proposed as early diagnostic markers and for the evaluation of cancer progression. Overall succinylation might represent a key therapeutic target in oncology [139].

Currently, the most likely enzyme exhibiting a role in histone succinylation is the deacetylase SIRT5 [25]. SIRT5 is predominantly a mitochondrial sirtuin that induces the urea cycle activation through the modulation of carbamoyl phosphate synthase (CPS1) [100, 140, 141] and promotes purine metabolism via urate oxidase [142]. SIRT5 exhibits a weak deacetylase activity but marked demalonylase, desuccinylase and deglutarylase activities. In light of this evidence, several approaches have been developed to target this promising therapeutic target. Recent researches report the structures and the biological function of SIRT5 inhibitors pointing out two compounds, a norharmane derivative and the small molecule derivate E)- 2- cyano- N- phenyl- 3- (5-phenylfuran- 2- yl)acrylamide, which shows, in a cell-based setting, high activity and isoenzyme selectivity [141, 143].

### Malonylation targeting

The enzyme most likely able to remove the malonyl group is SIRT5 [26]. Recently Zhang and colleagues provide evidence that the SIRT5 KO in mouse liver, dramatically increased Histone malonylation, enforcing the role of SIRT5 as a histone demalonylase [95]. The authors reported that levels of histone malonylation were higher in brain older mice pointing out that histone malonylation might exert ageing-associated nucleolar expansion, confirming overall, that peculiar epigenetic marks are related to aging associated pathways. SIRT5 appears to hold its deacetylating activity both in lysine Succinylation and in lysine malonylation. Similarly to succinylation, malonylation shows a crucial role in several signalling pathways and different pathologies. Functional analysis has underlined that malonyl-CoA, as a reactive thioester metabolite, might mark histone lysine influencing metabolic processes, stress responses and angiogenesis. Moreover, lysine malonylation is abundant in mitochondrial proteins and modulates crucial metabolic routes such as glycolysis and Lipolysis [144, 145].

### Glutarylation targeting

Evidences suggested that glutarylation is erased by Sirt7 a deacetylase preserving genome integrity and modulating DNA repair [146]. Sirt7 deficiency is related to an increased susceptibility to oxidative stress and genotoxic insults [147]. Notably, loss of function of Sirt7 with the following accumulation of H4K91glu is associated with chromatin decompaction [148]. We speculated that the most intriguing pathway that involves Histone glutarylation is the DNA damage pathway. Bao and colleagues reported that H4K91glu regulates chromatin structure and dynamics in response to DNA damage [71]. Here the authors suggested that Kglu orchestrates the mechanism of DNA repair by cooperating with Ksucc and Kac. In addition, they proposed that SIRT7, the deacetylase able to selectively remove Kglu, exhibiting a selective activity in modulating the kglu levels, might represent a promising molecular target [71]. In light of this evidence, it is reasonable that the use of SIRT7 inhibitors, in cancer cells presenting high levels of Kglu, might exert strategic anticancer effects. SIRT7 is a NAD^+^-dependent class III histone deacetylase (HDAC III) with a prevalent nuclear localization. Sirt7 is implicated in the regulation of the cell cycle, neoplastic transformation and metabolic homeostasis through the modulation of fatty acid metabolism, mitochondrial dynamic and lipogenesis [149].

The inactivation of SIRT7 rewires malignant phenotypes, impairs metastasis and increases response to therapy. In a recent study, Zhang and colleagues identified, by virtual screening, two compounds from the Chemdiv database, named 2800Z and 40569Z, that elicit the selective inhibition of SIRT7, promoting apoptosis [150]. As well, Kim and colleagues provide evidence that two suberoylanilide hydroxamic acid derivates exhibit a marked SIRT7 inhibition in a xenograft model of uterine sarcoma with concomitant impairment of tumour growth [151].

### Lactylation targeting

Regardless of the complex mechanism of lactylation installation, the delactylation process of lactyl-lysine in different types of proteins is mediated by Class I histone deacetylases (HDAC1-3) [74], which include human SIRT2 [152] and the E. coli homologous protein, CobB [78].

As a direct link between glucose metabolism (especially anaerobic glycolysis) and epigenetic regulations, lactylation may become a significant therapeutic target for diverse types of cancers and immuno-oncology in the future [16, 72, 153]. The important role of lactate in tumor microenvironment [154] further motivates the potential of lactylation as a therapeutic target in cancer treatment. To modulate the enzymatic pathways of lactylation, specific inhibitors targeting writer (lactylase) and eraser (delactylase) enzymes have been developed (i.e., HAT and HDAC inhibitors). On the other hand, to inhibit the non-enzymatic lactylation pathways, the biosynthesis of lactoylglutathione can be blocked by utilizing the inhibitors (such as BrBzGCp2) against glyoxalase 1 (GLO1) [155], which is the key enzyme converting methylglyoxal and glutathione to lactoylglutathione [156]. In summary, targeting the occurrence of lactylation in cells may become a promising therapeutic strategy, as HATs, HDACs, and GLO1 are specifically overexpressed in multiple types of cancers [157, 158].

### Glycation targeting

Even though it is challenging to prevent protein glycation due to its non-enzymatic feature, there are eraser enzymes identified that can actively remove the sugar molecules from the modified proteins [87, 88]. The non-cofactor-containing enzyme, DJ-1, was reported to act as a deglycase that can remove methylglyoxal and glyoxal from the modified histones and other proteins [159–163]. It was also shown to be a glyoxalase that can directly convert methylglyoxal to L-lactate in the presence of glutathione [164, 165]. In our previous studies, we have shown that a Ca2^+^-dependent enzyme, protein arginine deiminase 4 (PAD4), is able to specifically antagonize methylglyoxal-induced histone glycation, where it converts the methylglyoxal-modified arginine to citrulline to protect the target proteins from further glycation damage [159, 166]. Moreover, the adenosine triphosphate (ATP)-dependent enzyme, fructosamine 3 kinase (FN3K), was identified as another deglycase in human cells, which is able to convert the Amadori products back to lysines through the phosphorylation of fructoselysine residues [82, 91, 167]. Importantly, many of these glycation eraser enzymes are overexpressed in the cells of disease states, suggesting that they may serve as therapeutic targets. For example, DJ-1 and PAD4 are both highly overexpressed in many types of cancers (such as breast cancer), which are also referred to as oncoproteins [84, 166]. The anti-glycation and chromatin-protection roles of DJ-1 and PAD4 enable them to become promising targets for cancer treatment. Thus, specific inhibitors targeting DJ-1 and PAD4 have been developed for cancer therapies [168, 169] Similarly, inhibitors against FN3K have also been screened based on its enzymatic activity, which have great potentials to become drug leads in the future [170–172].

### Monoaminylation targeting

As the enzyme solely regulating histone monoaminylation, TGM2 is a significant druggable target for the treatment of diverse diseases [173]. Importantly, TGM2 is overexpressed in various kinds of cancers, making it a promising target for cancer therapies [174]. Based on its enzymatic activity, high-throughput screening assays have been developed to identify potent inhibitors against TGM2 [175]. Future in vivo tests and clinical trials may facilitate the application of TGM2 inhibitors for the therapy of cancer and other diseases.

## Discussion

The onset of cancer is the result of an intricate network of cooperating aberrations arising in several pathways as a consequence of both environmental and hereditary cues. In 2002, Hanahan and Weinberg provided a list of the cell “distinctive capabilities” acquired along by neoplastic transformation: the hallmarks of cancer, which were then updated in 2011. They include resistance to apoptosis, invasion and metastasis, sustaining proliferative signalling, metabolic reprogramming, neoangiogenesis, improvement of the replicative potential, sustaining immune evasion, genomic instability, evading growth inhibitors, and inflammation [176]. Although recent extraordinary advancements in medical oncology, the acquisition of resistance still represents the major Achilles’ hells in the therapy of cancer. Therefore, novel and selective therapeutic strategies need to be explored and implemented to improve treatments. Recently a tight correlation between cancer metabolism and epigenetic homeostasis has been reported and alteration of the epigenome landscape is often, if not always, involved in oncogenic transformation [177, 178]. In addition, it has been reported that in tumor tissues, single cell populations might exhibit specific patterns of histone modifications. The existence of an intrinsic epigenetic heterogeneity is clearly a notion of enormous impact in a translational context [179]. Overall, it seems now clear that cancer epigenetics modulates cellular behaviour including proliferation, apoptosis, invasion, and senescence. Therefore, profiling epigenetic architecture might shed light on molecular mechanisms underlying cancer phenotype providing a novel strategy to identify promising therapeutic targets.

As confirmed by several clinical trial aiming to identify peculiar druggable epigenetic marks currently “in itinere” [180, 181].

Here we have provided a survey of non-enzymatic modification correlating the metabolic fitness of cancer cells with epigenetic homeostasis. The most intriguing speculation is that the aberrant activation of a catabolic route will account for the overproduction of metabolites that ultimately will induce peculiar histone marks having profound consequences on cellular signalling. Therefore, in the context of personalized medicine, histone modifications as responsible for specific phenotypes might results crucial to define single patient responsiveness to a “precise” therapy. Moreover, it is reasonable that each modification might be selectively removed by a committed enzyme that might be “precisely” fought (Table 1).

**Table 1:** The table summarizes the main non-enzymatic modifications discussed in the review. We reported for each modification, the eraser accountable for the removal of non-enzymatic mark, and the drugs that exhibit a specific inhibitory activity

**NECM**	**Eraser**	**Drugs targeting the eraser enzyme**
Crotonylation	Histone deacetylates, Sirtuin 3 (SIRT3)	• 4’-bromo-resveratrol (4′-BR) [104] • 8-mercapto-3,7-dihydro-1H-purine-2,6-dione scaffold. [105] • BZD9Q [106] • Cambinol [107] • NƐ-acyl-lysine analogues [108] • 2-methoxyestradiol (2-ME) [109] • Butyrate [110] • Albendazole, [111] • 3-O-chloroacetyl-gagamine (A671) [112] • EX-527 [113] • LC-0296 [114]
Formylation	HDAC6	• Tubacin [118–120] • JBI-097 [121]
Propionylation	SIRT2	• Thioamide-containing sirtuin inhibitors [130] • Thiourea-containing sirtuin inhibitors [130]
β-ydroxybutyrylation	HDAC1 HDAC2	• Chidamide (CS055) [134] • Santacruzamate A, [135] • Thujaplicins, [136]
Succinylation	SIRT5	• Norharmane derivative [141] • 2- cyano- N- phenyl- 3- (5-phenylfuran- 2- yl)acrylamide, ty [143]
Malonylation	SIRT5	• Norharmane derivative and the [141] • 2- cyano- N- phenyl- 3- (5-phenylfuran- 2- yl)acrylamide, ty [143]
Glutarylation	SIRT7	• 2800Z [150] • 40569Z [150]
Lactylation	GLO1 HDAC1 HDAC2 HDAC3	• BrBzGCp2 [157, 158] • Inhibitors targeting HDAC1 [134, 135] • Inhibitors targeting HDAC2 [136] • Inhibitors targeting HDAC3 [22]
Glycation	DJ-1 PAD4	• Inhibitors targeting DJ-1 [168] • Inhibitors targeting PAD4 [169]

In this review, we propose a synopsis of peculiar patterns of Histones NECM affecting chromatin landscape in relation to metabolic fitness of cancer cells whose aberration has been related to cancer development and progression.

Therefore we also provide a parterre of peculiar targets that might be precisely fought in relation to the occurrence of peculiar Histones NECM providing a survey of drugs that exhibit a distinctive and selective inhibition of the enzyme involved in the removal of acylation marks underling those that exert significant antitumoral effects.

## Data Availability

All materials and data, supporting the conclusions of the manuscript, are included in the manuscript.

## Abbreviations

ACLY: ATP Citrate Lyase
ACSS2: Acyl-CoA Synthetase Short Chain Family Member 2
ACC: Acetyl-CoA carboxylase
βhb-CoA: β-Hydroxybutyryl-CoA
CPS1: Carbamoyl phosphate synthase
GCDH: Glutaryl-CoA dehydrogenase
HAT: Histone acetylase
HDAC: Histones deacetylases
HPTM: Histone post-translational modification
Kglu: Lysine glutarylation
Kβhb: Lysine β-hydroxybutyrylation
Kglu: Lysine glutarylation
Ksucc: Lysine succynilation
Kac: Lysine acetylation
Kpr: Lysine propionylation
KAT2A: Lysine acetyltransferase 2A
NECM: Non-enzymatic covalent modifications
PA: Propionic acid
PARP: Poly-ADP-ribose polymerase
PCC: Propionyl-CoA carboxylase
SCFA: Short-chain fatty acid
TCA cycle: Tricarboxylic acid cycle
TGM2: Transglutaminase 2
